# Foamy Virus Protein—Nucleic Acid Interactions during Particle Morphogenesis

**DOI:** 10.3390/v8090243

**Published:** 2016-08-30

**Authors:** Martin V. Hamann, Dirk Lindemann

**Affiliations:** 1 Institute of Virology, Medical Faculty Carl Gustav Carus, Technische Universität Dresden, 01307 Dresden, Germany; martin.hamann@tu-dresden.de; 2 CRTD/DFG—Center for Regenerative Therapies Dresden, Technische Universität Dresden, 01307 Dresden, Germany

**Keywords:** foamy virus, genomic viral RNA, *cis*-acting sequence, Gag biosynthesis, Pol biosynthesis, RNA encapsidation, capsid assembly

## Abstract

Compared with orthoretroviruses, our understanding of the molecular and cellular replication mechanism of foamy viruses (FVs), a subfamily of retroviruses, is less advanced. The FV replication cycle differs in several key aspects from orthoretroviruses, which leaves established retroviral models debatable for FVs. Here, we review the general aspect of the FV protein-nucleic acid interactions during virus morphogenesis. We provide a summary of the current knowledge of the FV genome structure and essential sequence motifs required for RNA encapsidation as well as Gag and Pol binding in combination with details about the Gag and Pol biosynthesis. This leads us to address open questions in FV RNA engagement, binding and packaging. Based on recent findings, we propose to shift the point of view from individual glycine-arginine-rich motifs having functions in RNA interactions towards envisioning the FV Gag C-terminus as a general RNA binding protein module. We encourage further investigating a potential new retroviral RNA packaging mechanism, which seems more complex in terms of the components that need to be gathered to form an infectious particle. Additional molecular insights into retroviral protein-nucleic acid interactions help us to develop safer, more specific and more efficient vectors in an era of booming genome engineering and gene therapy approaches.

## 1. Introduction

**Figure 1:**
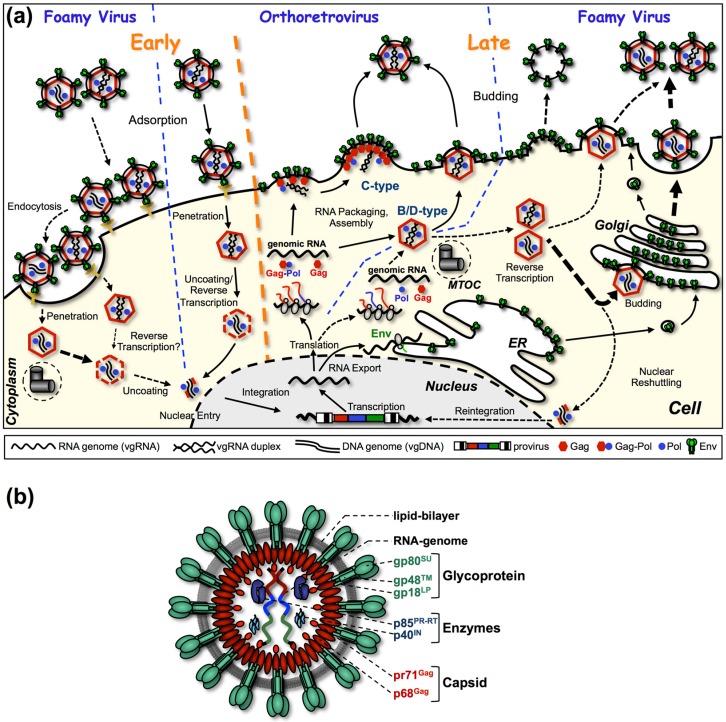
Foamy virus replication strategy and particle structure. (**a**) Comparative illustration of orthoretroviral and foamyviral replication cycles. The two main capsid assembly strategies utilized by retroviruses are illustrated in the center. C-type assembly comprises simultaneous assembly and budding processes at the plasma membrane. In contrast B/D type assembly is a two-step process with cytoplasmic preassembly of the capsid preceding budding at cellular membranes. Processes of entry and assembly of FVs, differing to that of orthoretroviruses, are illustrated to the left and right, respectively. FV specific replication steps are connected by dashed arrows, and replication steps specific to orthoretroviruses or common to both types of retroviruses are connected by solid arrows. MTOC: microtubule organizing center; ER: endoplasmic reticulum. (**b**) Schematic representation of the prototype FV isolate (PFV) particle structure. pr: precursor protein; p: protein; gp: glycoprotein. Panels (a) and (b) are adapted from [18].

Today spumaviruses, also called foamy viruses (FVs), comprise the only genus in the *Spumaretrovirinae* subfamily of retroviruses [1]. Becoming effective in 2005, a taxonomic reorganization of the *Retroviridae* family was issued as a consequence of accumulating evidence demonstrating that the replication strategy of FVs differs in several aspects from that of the other retroviral genera, which are nowadays grouped into the second, large subfamily of *Orthoretrovirinae* (Figure 1a) [1,2]. Interestingly, several of the “special replication features” of FVs bear some homology to characteristics of yet another virus family, the RNA genome encapsidating and reverse transcribing *Hepadnaviridae* [3].

In infected cells, FVs assemble new virions by a type B/D strategy, which separates capsid assembly and budding processes in a spatio-temporal manner (Figure 1a) [4]. In the case of FVs, first the Gag protein traffics to the centrosome where Gag preassembles into capsids [5]. In a second, separate step, budding of preassembled capsids of most FV species takes place at membranes of intracellular compartments and the cell surface. 

**Figure 2:**
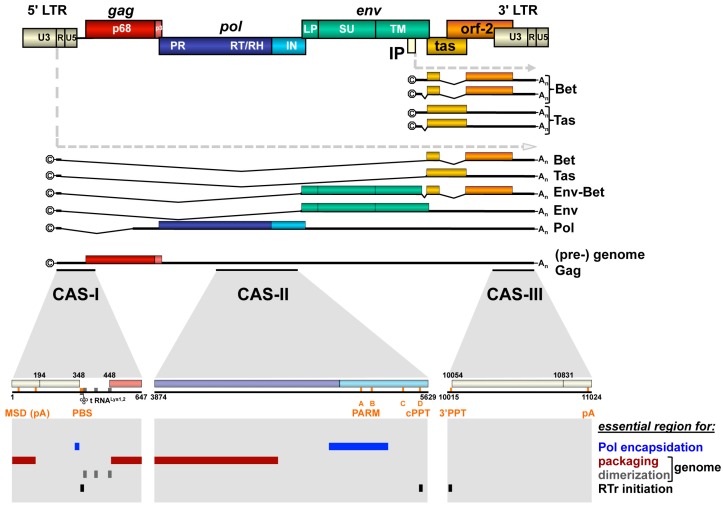
Foamy virus provirus organization, transcripts and essential *cis*-acting viral RNA sequence elements. Schematic illustration of the PFV proviral DNA genome structure with long terminal repeats (LTRs) and open reading frames (ORFs) indicated as boxes. For ORFs encoding Gag, Pol and Env precursor proteins, the regions encompassing the mature subunits generated by proteolytic processing are indicated by differential colors and are labeled accordingly. Spliced and unspliced viral transcripts originating from the LTR and internal promoter (IP) are schematically illustrated below and their respective coding capacity indicated to the right. *Cis*-acting sequence (CAS) elements localized within the full-length viral RNA genome, which are essential for viral replication, are indicated by black bars underneath the (pre-) genome RNA. Individual functionally important or essential RNA sequence motifs are marked in the enlarged individual CAS elements below. Numbers represent nucleotide positions of the viral PFV RNA genome (HSRV2 isolate). At the bottom, individual regions within the viral genomic RNA (vgRNA), which are essential for specific functions in viral replication as indicated to the right, are marked as differentially colored bars. U3: unique 3′ LTR region; R: repeat LTR region; U5: unique 5′ LTR region; ©: cap structure; A_n_: poly A tail; MSD: major splice donor; PBS: primer binding site; PARM: protease activating RNA motif; cPPT: central poly-purine tract; 3′ PPT: 3′ poly-purine tract; pA: polyadenylation signal; A-D: purine-rich sequence motifs A through D; RTr: reverse transcription. Adapted from [18].

Although assembly of FVs is similar to prototypic B/D type orthoretroviruses like Mazon-Pfizer monkey virus (MPMV) or mouse mammary tumor virus (MMTV), the FV replication cycle bears unique features not observed in any other retrovirus during these steps [2]. For example, FVs had to develop a unique mechanism for encapsidating their enzymatic proteins encoded by the *pol* open reading frame (ORF), since FV Pol is not expressed in an orthoretroviral-like Gag-Pol fusion protein but as a separate Pol precursor protein from a spliced mRNA (Figure 2) [6,7,8,9,10]. Furthermore, in a significant number (~5%–10%) of FV capsids, reverse transcription of the packaged viral genomic RNA (vgRNA) is already initiated in the capsids, preassembled at the centrosome. As a consequence, infected cells release both vgRNA- and viral genomic DNA (vgDNA)-containing FV particles, and reintegration of vgDNA into chromatin occurs (Figure 1a) [10,11,12,13]. Finally, budding of FV particles at cellular membranes requires expression of both the Gag as well as the Env protein, as FV Gag protein lacks a membrane targeting or membrane association domain [4,14,15]. For budding, preassembled FV capsids depend on a very specific interaction of Gag with the N-terminal cytoplasmic domains of the Env leader peptide subunit in order to initiate the budding process [16,17].

In summary, as a consequence of a unique Pol biosynthesis and the absence of membrane targeting domains in their Gag proteins, FVs evolved a special virus morphogenesis mechanism. Assembly of FV structural and enzymatic proteins as well as the viral genome encapsidation into capsids and their subsequent release is coordinated in a different manner than observed for other retroviruses.

In this review, we summarize the current knowledge on the FV genome encapsidation mechanism during virus morphogenesis. This includes a summary of the FV viral RNA genome, transcriptome and spliceosome and an overview of currently known viral *cis*-acting sequences including those of importance for RNA packaging or vgRNA dimerization. We also highlight the structural and functional differences of FV Gag and Pol proteins to their orthoretroviral counterparts with an emphasis on protein-nucleic acid interactions resulting in the packaging of viral and cellular RNAs into FV particles. Many of the graphics in this review refer to the prototype FV isolate (PFV), previously also known as human FV (HFV), since it is the best-studied FV species. However, throughout the review we also refer to other FV species if studies addressing the respective features discussed are available.

## 2. FV Transcription and *Cis*-Acting Viral Genomic RNA Sequences

### 2.1. Transcription 

Unlike most orthoretroviruses, the proviral DNA genome of FVs harbors an internal promoter (IP) element in addition to the canonical long terminal repeat (LTR) U3 region promoter (Figure 2) [19]. From the IP, which is located in the *env* ORF, spliced and unspliced subgenomic transcripts are generated. They encode the transcriptional transactivator Tas or the Bet protein, which is responsible for neutralization of cellular antiviral apolipoprotein B mRNA editing enzyme, catalytic polypeptide-like (APOBEC) proteins [20]. Expression from the LTR promoter is strictly Tas-dependent and results predominantly in transcripts encoding the FV structural and enzymatic proteins Gag, Pol and Env. However, although to a lesser extent, LTR-derived transcripts encoding the Tas and Bet protein can also be detected in infected cells.

### 2.2. *Cis*-Acting Sequences 

Like orthoretroviruses, the FV full-length vgRNA contains several *cis*-acting sequence (CAS) elements that are essential for its successful transmission to susceptible hosts and subsequent cellular replication of the virus (Figure 2). The 5′ and 3′ regions of the FV vgRNA, similar to their orthoretroviral relatives, contain elements essential for its reverse transcription into vgDNA as well as the subsequent integration of proviruses into host cell chromatin and their transcriptional regulation involving viral and cellular proteins. 

**Figure 3:**
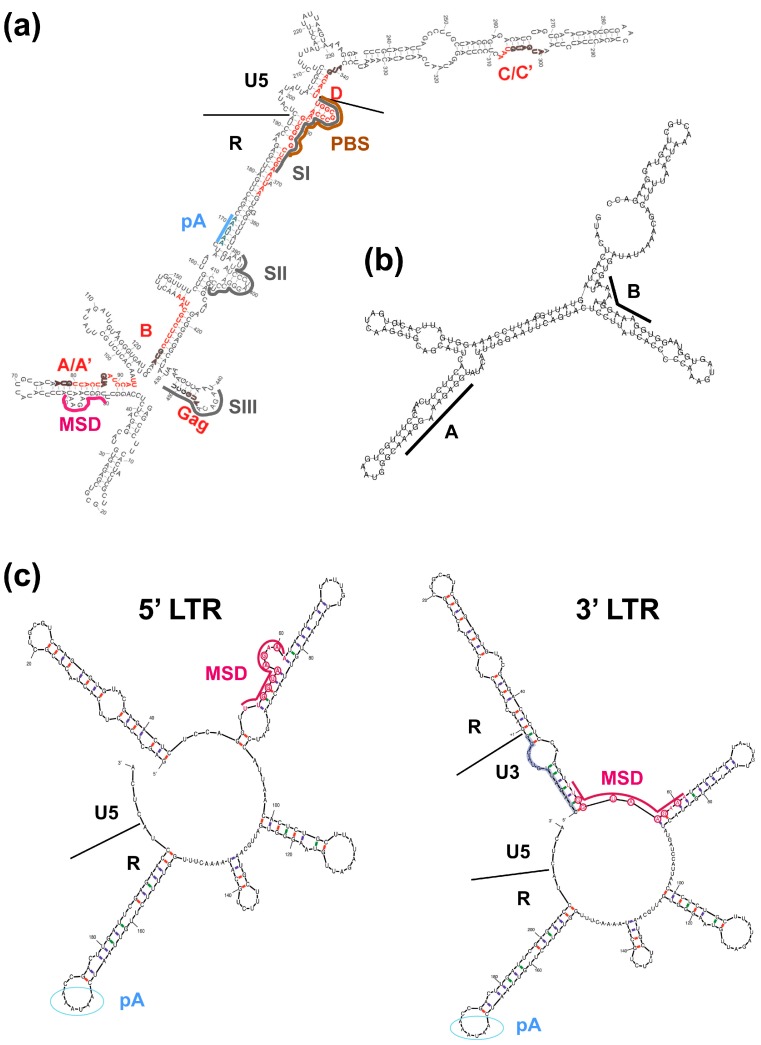
Secondary structure predictions of PFV *cis*-acting sequence elements. (**a**) Schematic representation of a partial PFV CAS-I sequence element structure (Reproduced and adapted, with permission, from [32]). Secondary structure, as predicted by the “M fold” program, of 5′ UTR sequences utilized for PFV *gag* translation by ribosomal shunting. The small open reading frames (sORFs) A–D in the leader are named and indicated by “thick red lines superimposed” on the structure. Major splice donor (MSD, violet), primer binding site (PBS, brown), polyadenylation signal (pA, blue) and dimer linkage site (DLS) sequence elements SI to SIII (dark gray) are marked by differentially colored lines. Boundaries of LTR R and U5 sequences are marked with black lines. (**b**) Schematic illustration of a partial CAS-II Pol encapsidation sequence (PES) sequence element structure (Reproduced and adapted, with permission, from [33]). RNA secondary structure, as predicted without constraints by the “RNAfold” program [34,35], of a 203 bp CAS-II sequence element, comprising the purine-rich sequences A and B of the protease-activating RNA motif (PARM), which is essential for PFV protease (PR) activation and Pol encapsidation. Purine-rich sequence elements A and B are marked by black lines. (**c**) Schematic representation of 5′ and 3′ PFV LTR sequences comprising the major splice donor (MSD) (Reproduced and adapted, with permission, from [31]). Secondary structure models, as predicted using selective 2′-hydroxyl acylation analyzed by primer extension (SHAPE) constraints by the “M fold” program [36,37,38], of a 198 bp sequence element of the PFV 5′ LTR (left) and a 211 bp sequence element of the PFV 3′ LTR (right). Illustrated are the differential folding of the MSD sequence, influencing its function in suppressing the pA signal by U1snRNP binding. Nucleotides complementary to the U1snRNA are circled (red) and the position of the pA signal is marked by blue circles. Boundaries of LTR R and U5 sequences marked with black lines.

The essential 5′ element of the FV vgRNA is termed CAS-I and in the case of PFV comprises nucleotides (nt) 1 to 645 (Figure 2 and Figure 3). It harbors the LTR R- and U5-regions, of which R is required for the strand switch after minus-strand strong stop DNA synthesis during reverse transcription, and U5 is recognized as substrate by the viral integrase in the linear double-stranded vgDNA facilitating proviral integration into host cell chromosomes. Furthermore, CAS-I contains additional functionally essential sequence elements located between LTR R-U5-region and the first 200 nt of the *gag* ORF (Figure 2). These include the primer binding site (PBS), which is complementary to the 3′ end of cellular transfer RNA (tRNA) Lys1,2 and required for minus-strand DNA synthesis initiation, as well as dimer linkage sequences (DLS) involved in genome dimerization (Figure 2 and Figure 3a) [21,22]. Three DLS sequence elements (SI to SIII) were characterized that contributed to dimerization of *in vitro* transcribed PFV CAS-I RNAs (Figure 3a) [23]. SI and SIII overlap with the PBS and *gag* translation initiation site. Mutation of these DLS elements, in context of proviral expression constructs, led to attenuated viral replication [21]. In contrast, alterations in the palindrome sequence of the SII element completely abolished viral replication. This suggests an important role of DLS SII in vgRNA dimerization and viral replication. Surprisingly, vgRNA encapsidation of DLS SII mutants was not reduced, indicating that dimerization is not a prerequisite for PFV vgRNA packaging. Moreover, CAS-I also includes the major splice donor (MSD) site used for splicing of subgenomic FV RNA transcripts derived from the 5′ LTR, which is located in the LTR R-region (Figure 2 and Figure 3a,c) [24,25]. Interestingly, its presence appears to be crucial for translation of the Gag protein from vgRNA and from expression constructs harboring authentic FV *gag* ORFs (see below) [26,27,28,29,30,31]. 

The CAS-III element located at the 3′ end of the FV genome harbors the LTR U3- and R-regions, which like the respective 5′ LTR sequences are involved in proviral integration and minus-strand switch during reverse transcription, respectively (Figure 2). Furthermore, the major transcription control elements recognized by cellular factors or the FV Tas protein, which are essential for the Tas-dependent proviral gene expression of FVs, are located in the LTR U3-region [19]. In addition, CAS-III includes the 3′ poly-purine tract (PPT) just upstream of the LTR U3-region, which serves as an additional initiation site for plus-strand DNA synthesis during reverse transcription.

Unique amongst retroviruses is the strict requirement of FVs for another essential *cis*-acting RNA sequence element, termed CAS-II [22,26,30,39,40]. It is located in the central part of the FV RNA genome and comprises a region of about 1.8 kb that encodes C-terminal domains of the Pol protein (Figure 2). CAS-II was found to be essential for replication of PFV, macaque simian FV (SFVmac) and feline FV (FFV), suggesting that this requirement for an additional CAS element is indeed a general feature of FVs. Four purine-rich sequence motifs (A–D) are located in the 3′ region of PFV CAS-II, one of which (D) is a perfect copy of the 3′ PPT and strongly conserved in all FV species (Figure 2) [41,42]. Purine-rich sequence motif D functions as a central poly-purine tract (cPPT), as found in lentivirus genomes, which serves as an additional plus-strand DNA synthesis initiation site during reverse transcription. However, unlike human immunodeficiency virus type 1 (HIV-1), FVs do not appear to contain a central flap region, which is important for efficient replication of HIV-1 genomes in non-dividing cells [42]. More importantly, it was recognized early on that CAS-II contains not only sequence elements important for viral genome packaging into newly formed capsids but also essential determinants required for Pol encapsidation, termed Pol encapsidation sequence (PES) [22,26,30,39,40]. A number of reports encompassing studies with PFV, SFVmac and FFV shaped the picture of CAS-II being a discontinuous element consisting of a 5′ part involved in genome encapsidation, a central region enabling Pol encapsidation, and the cPPT sequence motif located at its 3′ end (Figure 2) [22,26,39,40,43,44]. Wiktorowicz et al. [44] mapped the minimal elements of CAS-II for PFV and demonstrated that some internal genome sequences are functionally dispensable in the context of replication-deficient PFV transfer vector constructs, whose vgRNA is encapsidated into PFV particles generated by structural proteins expressed from respective Gag, Pol and Env packaging constructs in *trans*. Later Hartl et al. [33] discovered that the central PES element of CAS-II contains a sequence element, which includes the purine-rich motifs A and B and serves as a scaffold for PFV Pol dimerization required for viral protease (PR)-domain activation (Figure 2 and Figure 3b).

## 3. FV Gag Protein Biogenesis and Viral Genome Encapsidation

As in all retroviruses, the main structural building block of a fully assembled particle is composed of the *G*roup-specific *A*nti*g*en protein (Gag) [45]. The multifunctional Gag protein not only engages in protein-protein interactions, thereby forming the lattice of the assembled spherical capsid, but also plays an integral role in vgRNA packaging. Orthoretroviral Gag proteins are commonly characterized by their matrix (MA), capsid (CA) and nucleocapsid (NC) domains, which are located in that order from N- to C-terminus in the precursor Gag polyprotein. Upon maturation of the Gag precursor by multiple viral PR-mediated processing events during or shortly after virus release, the respective mature subunits, and in some cases, further peptides are generated [46]. Generally speaking, the MA domain mediates the Gag precursor membrane targeting and forms the outer shell of a mature orthoretroviral particle directly adjacent to the membrane. The CA domain composes the capsid core, whereas NC interacts tightly with the viral RNA genome. Both, MA and NC, were shown to have a certain affinity for nucleic acids, but it is NC, which is responsible for vgRNA selection and packaging [47]. 

**Figure 4:**
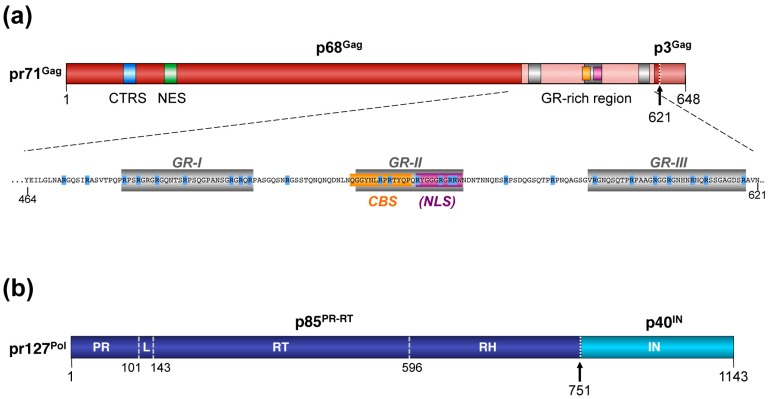
Schematic representation of the foamy virus Gag and Pol protein organization. (**a**) Schematic illustration of the PFV Gag protein organization and selected functional motifs. Several functional motifs of PFV Gag are highlighted in differentially colored boxes. The organization of the C-terminal GR-rich region and the amino acid sequence of the PFV Gag protein are shown in the enlargement below. Numbers indicate amino acid positions of the PFV Gag protein. The black arrow marks the cleavage site of pr71^Gag^ for processing into p68^Gag^ and p3^Gag^. Gray boxes represent the historically annotated GR boxes (GR-I to -III) within the domain, which is now referred to as the GR-rich region. Arginine residues are highlighted in blue. CTRS: cytoplasmic targeting and retention signal; NES: nuclear export signal; CBS: chromatin binding signal; (NLS): originally annotated putative nuclear localization signal. (**b**) Schematic illustration of the PFV Pol protein organization. Numbers indicate amino acid positions of the PFV Pol protein. The black arrow marks the cleavage site of pr127^Pol^ for processing into p85^PR-RT^ and p40^IN^. PR: protease domain; L: linker sequence; RT: reverse transcriptase domain; RH: RNase H domain; IN: integrase domain. Panels (a) and (b) are adapted from [18].

The overall structure of FV Gag proteins is different though, which directly raises the question of how FVs package their vgRNA (Figure 4a) [48,49]. Originally, MA, CA and NC domains were designated to the FV Gag precursor, however, the post-translational maturation of FV Gag is very limited compared with orthoretroviral Gag proteins and results in an immature capsid morphology of FVs (Figure 1b) [50,51]. Thus, the orthoretroviral domain terminology is no longer applied to FV Gag proteins.

### 3.1. Transcription and Translation 

FV Gag transcription and translation are regulated at different levels. 

First, at least three genetic elements were reported to influence expression of Gag, in context of its natural environment of the full-length vgRNA. The first one, found in the LTR R-region of the *gag* 5′ UTR sequences is the MSD, and was briefly mentioned above when discussing CAS-I (Figure 2 and Figure 3). The presence of the MSD appears to be crucial for the translation of the Gag protein from vgRNA or from expression constructs harboring the authentic 5′ UTR sequences and FV *gag* ORF [26,27,28,29,30,31]. Initially it was thought that the MSD is essential for nuclear export of full-length vgRNA encoding Gag [30]. However, it was recently shown that the MSD is involved in suppressing premature transcript termination by the PFV polyadenylation (pA) signal [31]. Like HIV-1 and murine leukemia viruses (MLV), the FV R-region also harbors the major pA signal, in the case of FVs found downstream of the MSD. This results in the presence of two pA signals in FV vgRNA, which necessitates a mechanism for suppression of the 5′ LTR pA site while allowing efficient utilization of the 3′ LTR pA site. Schrom et al. [31] nicely demonstrated that the MSD and differential RNA secondary structures of the 5′ and 3′ ends of the PFV RNA genome are essential determinants in this regulatory mechanism of FV transcription termination (Figure 3c). 

The second element, although poorly characterized, appears to be located within the *gag* ORF itself and inhibits expression of authentic *gag* ORFs in the absence of its 5′ UTR [13,28,29,30]. Its inhibitory effect can be neutralized by replacing the authentic FV *gag* 5′ UTR with a heterologous splice donor (SD) site or a complete intron sequence, but not by adding such sequences downstream of an authentic PFV *gag* ORF with 5′ UTR sequences lacking a SD site [13,28,29,30].

The third element is found in the 3′ UTR sequence of Gag encoding mRNAs and is one of the four poly-purine-rich sequences, element C, which was mentioned above during discussion of CAS-II (Figure 2). The study by Peters and colleagues [42], which analyzed the potential function of these sequences as cPPT elements, revealed an essential role for element C in Gag expression, although the underlying mechanism was not determined.

**Figure 5:**
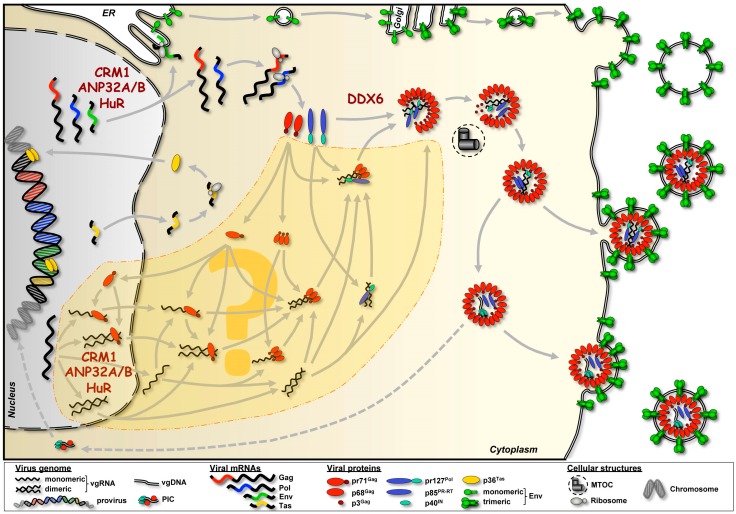
Pathways of FV RNA and protein trafficking resulting in capsid assembly and particle release. Please note that for simplicity budding of FV capsids at intracellular membranes was omitted. Known and putative trafficking pathways (marked by the dashed area with the question mark in the center) of viral RNAs from the nucleus into the cytoplasm to the capsid assembly site at the microtubule organizing center (MTOC) are shown. Putative RNA-protein interactions and assembly intermediates are depicted, as well as four different variants of vgRNA dimerization throughout the transport pathway to the assembly site illustrated. Known cellular cofactors involved in FV RNA trafficking or protein-RNA interaction are mentioned at the respective subcellular locations. Dashed arrows indicate the potential pathway leading to reintegration of vgDNA originating from preassembled capsids, which underwent reverse transcription prior to particle release [13]. CRM1: chromosomal maintenance 1; ANP32: acidic (leucine-rich) nuclear phosphoprotein 32 kDa; HuR: human antigen R; DDX6: DEAD-box helicase 6; PIC: pre-initiation complex.

Furthermore, nuclear export of PFV Gag encoding vgRNA is mediated by the karyopherin chromosomal maintenance 1 (CRM1) and also depends on the adaptor proteins acidic (leucine-rich) nuclear phosphoprotein 32 kDa (ANP32) A and B as well as interaction of the RNA binding protein human antigen R (HuR) with yet unknown elements in the PFV vgRNA (Figure 5) [52].

Finally, translation of the 71 kDa PFV Gag precursor protein (pr71^Gag^), encoded in full-length vgRNA containing the authentic *gag* 5′ UTR, appears to use a ribosomal shunting mechanism (Figure 3a) [32]. If and how this Gag translation strategy is coupled to mRNA export pathways utilized by FVs has not been investigated.

### 3.2. Post-translational Processing

The pr71^Gag^ precursor gets cleaved by the viral PR only once during particle morphogenesis, resulting in a large 68 kDa cleavage product (p68^Gag^) and a small p3^Gag^ peptide (Figure 4a). The processing patterns for simian and non-simian FV Gag proteins are identical, although the molecular weight of precursor and cleavage products differs slightly. Mature PFV particles, as they are found in the supernatant of infected cells, are composed of both pr71^Gag^ and p68^Gag^ forms at a ratio of 1:1 to 1:4 that is important for maximal viral infectivity (Figure 1b) [53,54,55,56,57]. FV Gag precursor processing is critical for capsid morphology and viral infectivity as viral mutants with an enzymatically inactive Pol PR domain are non-infectious and PFV Gag cleavage site mutants display a severe infectivity defect [4,56,57,58]. However, FV Gag processing seems to have no influence on vgRNA encapsidation. Furthermore, PFV Gag precursor cleavage during maturation appears to initiate, or at least enables completion of intra-particular reverse transcription (Figure 1a), which is another unique feature of FVs as mentioned earlier [54,59].

### 3.3. Trafficking and Intracellular Distribution 

Several peptide motifs within PFV Gag have been characterized that influence its intracellular distribution and/or trafficking, although some of their functions in viral replication are not fully understood or are discussed controversially (Figure 3) [60,61]. 

A cytoplasmic targeting and retention signal (CTRS) and a nuclear export signal (NES) are located at the N-terminus of PFV Gag [60,61]. The PFV CTRS shares sequence homology to a respective sequence motif of MPMV and is thought to be responsible for targeting newly translated PFV Gag to the cellular centrosome [60]. Mutation of the CTRS abolishes intracellular capsid assembly and leads to a localization of mutant Gag that is predominantly nuclear [5]. For the NES, a function in export of nuclear localized Gag was reported, and a potential role in nuclear export and selective encapsidation of vgRNA was proposed [61]. However, another study failed to detect any involvement of PFV Gag in nuclear export [52].

The temporary nuclear localization of Gag has long been recognized as a hallmark in FV infected cell cultures [62,63]. A putative nuclear localization signal (NLS), identified in the glycine—arginine (GR)-rich C-terminal domain of Gag (Figure 4a), was originally reported to be responsible for the transient trafficking of nascent Gag through the nucleus [64,65]. However, later on, a live-cell imaging analysis study, using fluorescent protein tagged PFV Gag, failed to demonstrate any signs of active import into interphase cell nuclei [66]. Instead, this study nicely demonstrated that authentic PFV Gag nuclear localization is only occurring upon nuclear membrane breakdown during cellular mitosis. Notably, only PFV Gag tagged with a heterologous simian virus 40 (SV40) NLS was actively imported into interphase nuclei. 

During mitosis, authentic PFV Gag attaches to host cell chromatin via a chromatin binding signal (CBS) and remains in the nucleus after cell division [66,67]. The CBS identified earlier by Tobaly-Tapiero and colleagues [68] resides just upstream of the original putative NLS and mediates binding to H2A/H2B core histones (Figure 4a). Whether chromatin tethering of nascent Gag in FV infected cells represents a dead end trafficking event, or whether some Gag is subsequently exported (with or without vgRNA) via the characterized N-terminal NES, was not investigated by Müllers and colleagues [66] and needs to be addressed in future studies. It was suggested, that the PFV Gag CBS has a function during initial stages of viral replication by tethering Gag, which most likely is a component of the preintegration complex, to mitotic chromosomes [66,69]. Recent results confirm this notion, and demonstrate that the PFV Gag CBS influences the integration site profile (P. Lesbats and P. Cherepanov, personal communication).

### 3.4. Nucleic Acid Interactions 

In regard to Gag interacting with nucleic acids, the C-terminal region of the protein stood in the limelight early on. Whereas in the orthoretroviral Gag protein, single or double cysteine-histidine zinc finger motifs (Cys-X2-Cys-X4-His-X4-Cys) localized in the C-terminal NC domain were characterized to be essential for vgRNA recognition and selective packaging, FV Gag proteins lack these motifs [45]. Instead, they contain C-terminal glycine-arginine-rich patches, referred to as GR boxes, which are considered to be functionally equivalent (Figure 4a). Historically classified, PFV and other simian FV Gag proteins contain three GR boxes, GR-I to -III [64]. In contrast, non-primate FV Gag proteins inherit no apparent clustering of glycine-arginine residues [70,71]. Among all GR boxes, GR-II shows the highest sequence conservation, whereas GR-I and GR-III are considered to be more closely related to each other and functionally interchangeable [72].

Several early studies aimed to identify the individual functions of each GR box in PFV [64,65,73]. Initial studies employing *in vitro* binding assays with recombinant C-terminal PFV Gag protein domains and nucleic acids assigned a function in vgRNA binding to GR-I [65]. Later, phenotypic characterization of proviral mutants also suggested roles for GR-I in Pol packaging [73,74]. Lee and colleagues characterized GR box I mutants that packaged vgRNA at wild-type level but apparently failed to encapsidate Pol [74]. Additional complexity was introduced by a comprehensive study encompassing all three GR boxes using a four-component PFV vector system [72]. Deletion or substitution of any individual GR box in PFV Gag resulted in a drastic loss of infectivity (mainly due to reduced intra-particular reverse transcription) and Gag ∆GRI mutant particles had severely malformed capsids. In contrast, particle release and Pol encapsidation of all individual GR box mutants was not affected and only moderate reduction in vgRNA packaging was detectable. The reasons for these deviating results of the different studies are not completely clear but may originate from using different study systems (recombinant Gag protein domains, proviral and four-component vector systems) as well as introducing different GR box alterations (truncations, deletions, substitutions).

Different functions were attributed to GR box II that changed significantly over time. This is mainly due to the fact that the sequence element, which was originally designated as GR-II, comprises the CBS- and formerly annotated NLS element, as discussed in detail above. However, a distinct functional role of GR-II amongst the three GR boxes of PFV Gag can be deduced, since it cannot be functionally complemented by GRI or GRIII, whereas GRI and GRIII seem functionally interchangeable [72].

Taken together, the analysis of GR box functions led to variable results regarding the different roles of individual GR boxes in foamy virus replication. In some cases, seemingly contradicting findings led to different conclusions. Additionally, it is likely that sequences flanking the GR boxes, some of which are conserved in FV Gag proteins, are equally important for either vgRNA encapsidation or other steps in the replication cycle. This indicates that a strict focus on individual GR boxes hampers our understanding of the FV Gag protein. In line with this, by deleting all three GR boxes simultaneously or substituting almost all arginine residues in the C-terminus of PFV Gag by alanine (Figure 4a), we recently showed that it is the cooperative action of the arginine residues and not the function of an individual GR box that mediates binding of viral and cellular RNAs [75]. The significantly reduced particle release and aberrant capsid morphology of these Gag mutants unable to bind RNAs suggest that RNA (of viral and/or cellular origin) is an essential structural component in capsid assembly and release, a feature that was already observed for other retroviruses [75,76]. These recent results encourage envisioning the C-terminus of FV Gag as a GR-rich domain, wherein the positively charged arginine residues enable binding of RNAs in a cooperative manner leading to their encapsidation into newly formed capsids. However, further conserved and functionally distinct motifs located within this region, such as the CBS, may be essential for other steps of the FV replication cycle.

All previous studies on the role of FV Gag in vgRNA encapsidation and even the newly emerging scheme of an RNA binding Gag C-terminus, however, do not provide any details about the mechanisms resulting in the specificity and selectivity of FV vgRNA packaging. How do FVs ensure that two copies of full-length vgRNA end up in an emerging particle? The C-terminal arginine residues in PFV Gag bind and encapsidate both viral and cellular RNAs and vgRNA dimerization appears to be non-essential for vgRNA packaging [21,75]. Unfortunately, a Gag mutant that does package vgRNA but no cellular RNAs (or vice versa) has not been described yet.

In summary, it seems reasonable that the lack of cysteine-histidine motifs in FV Gag calls for another alternative packaging mechanism compared to orthoretroviruses and thus once more highlights the distinct features of FV to orthoretroviruses. Addressing the open questions may reveal a so far unknown mechanism of how viruses assure their replication and at the same time solve an eminent problem with an astonishingly small repertoire of RNA sequences and proteins.

## 4. FV Pol Biogenesis and Viral Genome-Dependent Encapsidation

### 4.1. Transcription and Translation 

The biogenesis of the FV Pol protein is quite different from that of orthoretroviruses. As mentioned above, FVs translate Pol as a separate pr127^Pol^ precursor protein from a singly spliced mRNA and not as a Gag-Pol fusion protein (Figure 2 and Figure 5) [6,7,8,10]. The level of Pol expression in FV infected cells appears to be regulated by the amount of singly-spliced *pol* mRNA, generated using a suboptimal splice site located upstream of the *pol* ORF [77]. Although not investigated in detail, results of Bodem and colleagues [52] on nuclear export of PFV Gag encoding vgRNA suggest that the same CRM1-dependent pathway is used for singly-spliced *pol* mRNA (Figure 5). 

Remarkably, FV replication is compatible with expression of FV Pol in an orthoretroviral-like manner as a Gag-Pol fusion protein. However, when PFV *gag* and *pol* ORFs are artificially genetically fused or connected by a HIV-1 ribosomal frameshift sequence, proteolytic processing between Gag and Pol domains of the generated Gag-Pol fusion proteins needs to be ensured for functional compatibility with viral replication [78,79,80]. This indicates, that unlike orthoretroviruses PFV particle release and infectivity tolerates larger differences in relative cellular Gag/Pol levels.

### 4.2. Posttranslational Processing and Nucleic Acid Interactions 

Maturation of the natural FV Pol precursor protein is also quite unique (Figure 4b). Only a single processing site within PFV pr127^Pol^ is utilized during autocatalytic proteolysis by the viral encoded PR, which results in generation of two mature subunits, an N-terminal subunit (p85^PR-RT^ in the case of PFV) and a C-terminal subunit (p40^IN^ in the case of PFV) as found in released FV particles (Figure 1b) [6,7,10,81]. Unlike orthoretroviruses, FV particles do not contain a separate PR subunit after completion of Pol precursor maturation [46,82]. Instead the mature PFV p85^PR-RT^ subunit serves as a multi-domain protein where an N-terminal PR domain is covalently connected via a linker sequence with a central RT- and a C-terminal RNase H domain (Figure 4b) [83]. Similar to orthoretroviruses the PFV integrase constitutes a separate subunit, p40^IN^, harboring only the integrase domain [81]. Ultimately the p40^IN^ subunit tetramerizes and associates with the linear, double-stranded viral DNA genome, generated by reverse transcription of the encapsidated PFV RNA genome, to form the intasome complex, which mediates proviral integration [84].

Autocatalytic processing of the pr127^Pol^ precursor is thought to occur predominantly during or shortly after encapsidation into newly assembled capsids [80]. PFV PR is only active as dimer [85], and two not mutually exclusive mechanisms for activation of the PFV PR by transient dimerization are discussed. The first involves pr127^Pol^ dimerization through interfaces within the C-terminal IN domain. The second hypothesis proposes dimerization of pr127^Pol^ and/or p85^PR-RT^ on RNA secondary structures encoded in the CAS-II element of the viral genome as the main trigger for PR activation [33,86].

All experimental evidence accumulated until now strongly supports a Pol encapsidation mechanism that is linked to viral genome packaging, although Gag-Pol protein-protein interactions may be involved as well, but seem to not be alone sufficient [28,39,43,74]. In our current model of Pol encapsidation the viral genome serves as a scaffold connecting the Pol precursor protein to the assembling capsid structure. It requires sequence motifs or secondary structures encoded within the FV RNA genome that enable selective interactions with both Gag and Pol proteins. From the analysis and characterization of essential *cis*-acting viral sequences summarized above, it appears that Pol interacts with specific PES elements contained within CAS-I and CAS-II of the FV genome [28,30,39,42,44]. The PES elements can be clearly separated physically and functionally from other RNA elements important for genome encapsidation by the Gag protein, as they are not essential for this process (Figure 2) [42,44]. However, mechanistic and molecular details as well as spatio-temporal characteristics of the FV Pol encapsidation process are largely unknown. Interestingly, only the pr127^Pol^ precursor but not the mature p85^PR-RT^ and p40^IN^ is efficiently encapsidated into assembling PFV capsid structures [43,68]. This may suggest that in context of the Pol precursor both the PR-RT as well as the IN domain are involved in direct interactions with CAS elements and/or the Gag protein, which are essential for Pol encapsidation. Though, it is not known whether the PR-RT and IN domain within the Pol precursor fold similarly to the mature PR-RT and IN subunits. Furthermore, only for the p85^PR-RT^ subunit RNA binding activities have been described [87,88]. Alternatively, oligomerization of the Pol precursor, which allows Pol encapsidation, through regions within the C-terminal IN domain may be necessary for or enhance the interaction of the N-terminal PR-RT domain with the PES elements within the vgRNA [33,86].

## 5. Cellular Cofactors Interacting with FV RNAs

Our knowledge about cellular cofactors involved in FV protein-vgRNA interactions and vgRNA encapsidation is extremely limited. As mentioned above, a complex comprising the karyopherin CRM1, the adaptor proteins ANP32A and B, and the RNA binding protein HuR was shown to be responsible for nuclear export of Gag [52] (Figure 5). Probably, it also mediates nuclear export of Pol and Env encoding viral mRNAs, and thereby enables translation of all the viral structural and enzymatic proteins in the cytoplasm. Structural differences in Gag encoding mRNA and vgRNA packaged into viral particles have yet to be identified, suggesting they are identical. Although, to the best of our knowledge this has not been addressed directly until now [52]. It is therefore very likely that the same CRM1-dependent nuclear RNA export pathway is employed for vgRNA transport into the cytoplasm, and hence contributes indirectly to vgRNA packaging (Figure 5). 

The only additional co-factor described to date of being involved in vgRNA encapsidation, is the DEAD-box RNA helicase DDX6 [89] (Figure 5). Endogenous DDX6 was found to be redistributed in PFV infected cells to the capsid assembly site at the centrosome. Knockdown of DDX6 via siRNA led to reduced vgRNA encapsidation, and the ATPase/helicase activity of DDX6 was required for viral replication. Further mechanistic details of the DDX6 involvement in vgRNA packaging remain to be identified, since neither a stable interaction with PFV Gag nor an encapsidation in viral particles was detectable. Therefore, an indirect contribution of the DDX6 enzymatic function and/or the unwinding activities, in transiently moderating the conformation of vgRNA and/or vgRNA-Gag ribonucleoprotein complexes, is thought to facilitate incorporation of the viral RNA into particles [89].

## 6. Conclusions and Perspectives

In the past two decades the molecular details of selective vgRNA encapsidation in orthoretroviruses, pioneering in MLV and HIV-1, became fully appreciated. However, this appreciation only emerged after the eminent role of secondary RNA structures in the retroviral genome was recognized, and laborious molecular and cellular analysis of RNA and Gag interactions were conducted [90,91,92,93]. 

In FV research, orthoretroviral relatives almost always serve as primary reference models for investigating the molecular details of their replication cycle. Whereas the knowledge obtained from many studies on orthoretroviruses helped to understand the FV biology, the differences in their replication strategies are now more and more challenging to expand the current picture on FV replication. Here we provided a condensed account on the similarities, and more importantly of the differences, of the FV Gag and Pol biosynthesis as well as the essential *cis*-acting genome sequences compared to orthoretroviruses. With special emphasis on Gag and Pol interacting with nucleic acids during particle morphogenesis, we conclude that the way we picture FV Gag to engage, bind and package RNA has changed over time. Abandoning the view of individual GR boxes having functions in RNA interactions, and focusing more on a general RNA-binding Gag C-terminus, now paves the route in addressing further mechanistic and molecular details of vgRNA encapsidation.

To fully comprehend the picture of vgRNA packaging, we also need to address the other side of the process: understanding which vgRNA motifs are essential to specifically bind Gag. As mentioned above, several reports highlight sequences in CAS-I and II within the FV genome important for vgRNA encapsidation (Figure 2). However, a more detailed analysis is required to fully understand the molecular mechanism behind. When and where do Gag and vgRNA as well as cellular RNAs interact (Figure 5)? Are there differences in the RNA binding features of monomeric versus oligomeric FV Gag, or are there yet uncharacterized Gag peptide motifs that might be essential for the selective vgRNA packaging? Where is dimerization of the vgRNA taking place, and is it occurring prior to interaction with the FV Gag protein, or not (Figure 5)? If not, does FV Gag possess chaperone function, as reported for some orthoretroviral Gag proteins or their cleavage products, and does it thereby contribute to or facilitate vgRNA dimerization and/or refolding? To further pinpoint and characterize individual sequence motifs or secondary structures in the vgRNA involved in these processes, is therefore a mandatory task. Possibly delineating vgRNA selection and encapsidation in regard to Gag translation and oligomerization, together with the spatio-temporal information of genome dimerization and initiation of reverse transcription, should be the ultimate goal that is desirable to be achieved (Figure 5).

As outlined above, the expression of FV Pol from an individual transcript is not shared by orthoretroviruses, but observed for hepadnaviruses, which share some replication characteristics with FVs [2,3]. This unique FV feature within the retrovirus family increases the complexity of particle morphogenesis, and instantaneously poses further key questions: how is Pol encapsidated into nascent particles, if not directly interacting with Gag? And how does the virus ensure that at least four Pol molecules end up in the particle to form a fully functional tetrameric intasome? The current hypothesis, of vgRNA binding both Gag and Pol at distinct sequences and thus functioning as a bridge, is supported by identification of vgRNA sequences associated with specific FV Pol binding. This “bridging hypothesis” is further supported by the observation that only the Pol precursor is efficiently encapsidated into nascent FV particles. However, the sequential order of Gag and Pol interactions with vgRNA has not yet been determined (Figure 5). Perhaps engagement of vgRNA by FV Pol is involved in selective genome encapsidation by FV Gag proteins, a mechanism that is utilized by hepadnaviruses [3]. However, until now no essential function of FV Pol in vgRNA encapsidation has been observed.

Elucidating the details of FV RNA packaging is not a mere scientific footnote. It might help to improve FV vector design, which in the recent past showed promising properties for application in gene therapy, and bears potential to safeguard genome engineering techniques by its beneficial integration profile.
